# Supplementary material for: Global Epidemiology of Invasive Infections by Uncommon Candida Species: A Systematic Review
Source: J Fungi (Basel). 2024 Aug 7;10(8):558. doi: 10.3390/jof10080558 (PMC11355942; doi:10.3390/jof10080558)
Supplement: Supplementary file 1 [file jof-10-00558-s001.zip › Supplementary Material Figures.pdf]

*Systematic Review*

# Global Epidemiology of Invasive Infections by Uncommon *Candida* Species: A Systematic Review

**Sandra Pinho [1], Isabel M. Miranda [2] and Sofia Costa-de-Oliveira [3,4,*]**

[1] Faculty of Medicine, University of Porto, 4200-319 Porto, Portugal; sdrpinho@gmail.com
[2] Cardiovascular R&D Centre UnIC@RISE, Department of Surgery and Physiology, Faculty of Medicine, University of Porto, 4200-319 Porto, Portugal; imiranda@med.up.pt
[3] Division of Microbiology, Department of Pathology, Faculty of Medicine, University of Porto, 4200-319 Porto, Portugal
[4] Center for Health Technology and Services Research—CINTESIS@RISE, Faculty of Medicine, University of Porto, 4200-319 Porto, Portugal
* Correspondence: sqco@med.up.pt

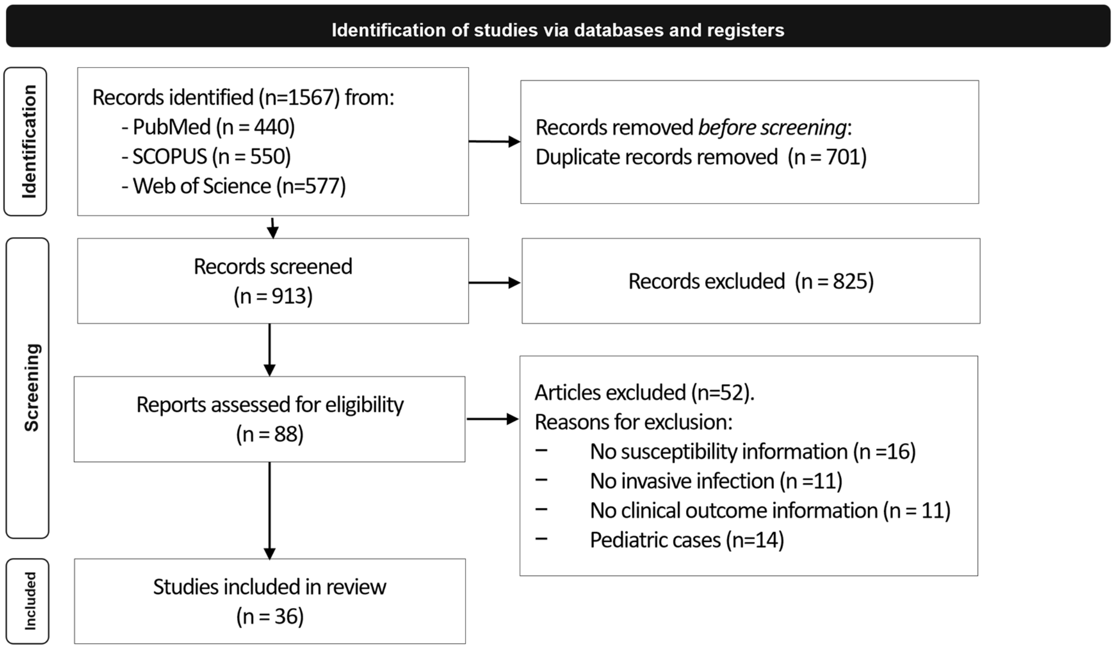


**Figure S1:** PRISMA flow diagram summarizing the search results.

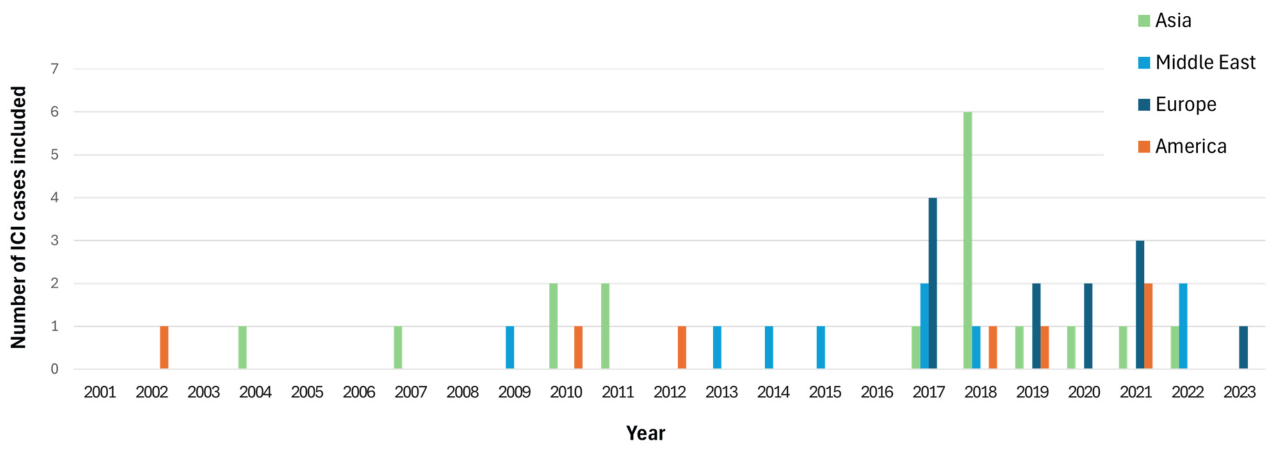


**Figure S2:** Number of included cases of uncommon *Candida* invasive infections according to year and localization

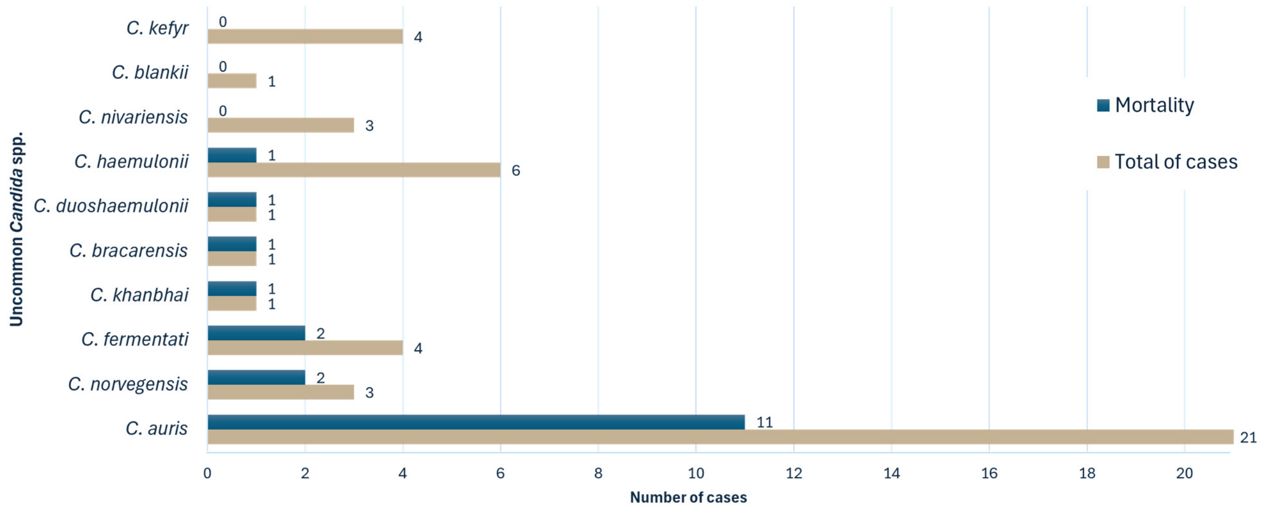


**Figure S3:** Mortality associated with invasive infection according to each uncommon *Candida* spp.

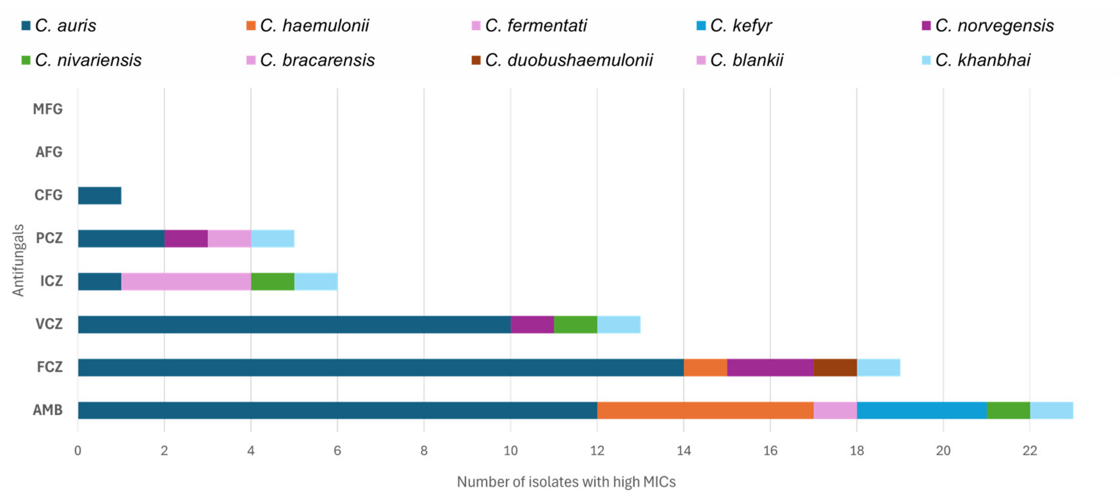


**Figure S4:** Number of isolates with high MICs according to the type of species.
