# Supplementary material for: Global Epidemiology of Invasive Infections by Uncommon Candida Species: A Systematic Review
Source: J Fungi (Basel). 2024 Aug 7;10(8):558. doi: 10.3390/jof10080558 (PMC11355942; doi:10.3390/jof10080558)
Supplement: Supplementary file 1 [file jof-10-00558-s001.zip › Supplementary Material Tables.pdf]

Systematic Review

Global Epidemiology of Invasive Infections by Uncommon *Candida* Species: A Systematic Review

Sandra Pinho [1], Isabel M. Miranda [2] and Sofia Costa-de-Oliveira [3,4,*]

[1] Faculty of Medicine, University of Porto, 4200-319 Porto, Portugal; sdrpinho@gmail.com
[2] Cardiovascular R&D Centre UnIC@RISE, Department of Surgery and Physiology, Faculty of Medicine, University of Porto, 4200-319 Porto, Portugal; imiranda@med.up.pt
[3] Division of Microbiology, Department of Pathology, Faculty of Medicine, University of Porto, 4200-319 Porto, Portugal
[4] Center for Health Technology and Services Research—CINTESIS@RISE, Faculty of Medicine, University of Porto, 4200-319 Porto, Portugal
* Correspondence: sqco@med.up.pt

Table S1: Description of the PICO anagram

PICO strategy	Elaboration of the study strategy
Population	Adults (age ≥ 18 years old) diagnosed with uncommon *Candida* spp. invasive infection
Intervention	Epidemiology of uncommon *Candida* spp. invasive infections
Comparison	Not applicable to this study
Outcome	Diagnosis, clinical, and geographic information

Table S2: Characteristics of patients with invasive infections by uncommon *Candida* species.

	Author/Year	Country	Candida species	G	Age (years)	Hospital stay (days)	Death	Comorbilities	Site of isolation	Subsquent infection type	Antifungal treatment	MV	IC	Previous broad-spectrum ATB	PAT	Other infections	Microorganism
America	Biagi, M. J. et al., 2019 [65]	USA	*C. auris*	M	54	≥100	No	Recurrent and recent SEPSIS<br>Chronic tracheostomy<br>Chronic indwelling<br>Tubecolostomy	Blood	Candidemia	MFG<br>PCZ<br>FCZ	Yes	Yes	Yes	Yes	candiduria	*C. auris*
	Parra-Giraldo, C. M. et al., 2018 [112]	Colombia	*C. auris*	M	74	17	Yes	Neuroleptic malignant syndrome<br>Rhabdomyolysis<br>Acute kidney failure<br>Major depression<br>Hypertension<br>Mitral valve replacement<br>Hypothyroidism	Blood	Candidemia	AFG	No	Yes	Yes	No	Nosocomial pneumonia	*Klebsiella pneumoniae*
	Kollu, V. S. et al., 2021 [52]	USA	*C. blankii*	M	63	NI	No	Hypertension<br>Dislipidemia<br>Diabetes mellitus<br>DRGE<br>AVC<br>SEPSIS recently	Blood	Endocarditis	AMB<br>MFG	NI	Yes	Yes	Yes	Bacteriemia	*Staphylococcus lugdunensis*
	Warren, T. A. et al., 2010 [53]	Canada	*C. bracarensis*	M	50	53	Yes*	Chronic lymphocytic leukemia<br>Bone marrow transplant 7 years previously<br>Graft-versus-host disease	Blood	Candidemia	CFG	NI	Yes	Yes	NI	Yes	*Klebsiella pneumoniae*
	Rodero, L. et al., 2002 [50]	Argentina	*C. haemulonii*	M	83	NI	No	Megaloblastic anemia	Blood	Candidemia	NI	No	No	Yes	No	No	No
	Pérez-Lazo, G. et al., 2021 [51]	Peru	*C. haemulonii*	F	72	43	No	Congenital polycystic kidney<br>Liver disease<br>End-stage renal disease on Hemodialysis	Purulent liver collection	Hepatic abscess	CFG	No	Yes	Yes	No	No	No

	Author/Year	Country	Candida species	G	Age (years)	Hospital stay (days)	Death	Comorbilities	Site of isolation	Subsequent infection type	Antifungal treatment	MV	IC	Previous broad-spectrum ATB	PAT	Other infections	Microorganism
	Almeida-Jr, J. N. et al., 2012 [49]	Brazil	*C. haemulonii*	M	26	8	Yes	Ovarian carcinoma	Blood	Candidemia	NI	NI	Yes	Yes	No	No	No
	Corpus, K. et al., 2004 [67]	USA	*C. kefyr*	M	64	24	No	Colon cancer	Pleural fluid	Pulmonary infection	FCZ<br>AMB<br>VCZ	NI	Yes	Yes	Yes	Yes	*Vancomycin-resistant enterococcus faecium*
**Asia**	Tsai, Y. T. et al., 2022 [32]	Taiwan	*C. auris*	M	64	42	No	Diabetes mellitus<br>Ischaemic stroke	Blood	Candidemia	AFG	yes	yes	yes	yes	Nosocomial infection	NI
**Asia**	Das, S. et al., 2018 [61]	India	*C. auris*	F	58	46	No	Hypertension<br>Left ventricular failure<br>Bronchitis<br>Acute renal failure<br>Spinal muscular dystrophy	Blood	Candidemia	FCZ	Yes	Yes	Yes	No	Yes	*Acinetobacter species*
**Asia**			*C. auris*	M	50	25	No	Alcoholic<br>Chronic smoker	Blood	Candidemia	FCZ	Yes	No	Yes	No	Tracheal aspirate	*Acinetobacter species*
**Asia**			*C. auris*	F	26	25	No	Puerperal sepsis<br>Acute kidney injury<br>Multiple organ dysfunction syndrome	Blood	Candidemia	NI	Yes	Yes	Yes	No	Yes	*Klebsiella pneumoniae*
**Asia**	Vasilyeva et al., 2018 [55]	Russia	*C. auris*	F	88	43	Yes	acute stroke	Blood	Candidemia	AFG	Yes	Yes	Yes	NI	NI	
**Asia**	Lee, W. G. et al., 2011 [35]	South Korean	*C. auris*	M	74	79	Yes	Layngeal squamous cell carcinoma Laryngectomy	Blood<br>Catheter tip	Candidemia	FCZ<br>AMB	NI	yes	NI	NI	no	No
**Asia**	Xie, O. et al., 2020 [56]	Vietnam	*C. duobushaemulonii*	M	85	9	Yes	Non-insulin dependent diabetes<br>IgA nephropathy	Blood	Candidemia	NI	NI	Yes	Yes	NI	Yes	*carbapenem resistant Klebsiella pneumoniae*
**Asia**	Konuma, T. et al., 2017 [59]	Japan	*C. fermentati*	M	68	100+	No	Leukaemia<br>Allogeneic hematopoietic cell transplant	Blood	Candidemia	AMB<br>5-FC	NI	Yes	Yes	Yes	No	N/A
**Asia**	Morita, K. et al., 2018 [34]	Japan	*C. fermentati*	M	59	35	No	Leukaemia<br>Cord blood transplantation	Blood<br>Bronchial lavage fluid	Candidemia	AMB	No	Yes	Yes	Yes	No	No

	Author/Year	Country	Candida species	G	Age (years)	Hospital stay (days)	Death	Comorbidities	Site of isolation	Subsequent infection type	Antifungal treatment	MV	IC	Previous broad-spectrum ATB	PAT	Other infections	Microorganism
			C. fermentati	F	63	45	Yes	Leukaemia<br>Cord blood transplantation	Blood	Candidemia	MFG<br>AMB	No	Yes	Yes	Yes	No	No
			C. fermentati<br>*C. famata*	M	70	24	Yes	Leukaemia<br>Cord blood transplantation	Blood<br>Bronchial lavage fluid	Candidemia	AMB<br>MFG<br>AMB	Yes	Yes	Yes	Yes	No	No
	Ruan, S. Y. et al., 2010 [60]	Taiwan	*C. haemulonii*	M	79	NI	No	Hypertension<br>Pneumonia with respiratory failure<br>Pneumothorax	Blood	Candidemia	FCZ	Yes	No	No	No	No	No
			C. haemulonii	M	85	25	No	Advanced rectal adenocarcinoma<br>Surgery	Blood	Candidemia	FCZ<br>MFG	Yes	Yes	NI	NI	No	No
	Kim, S. et al., 2011 [63]	South Korean	*C. haemulonii*	M	67	55	No	Cerebral Infarction<br>Tracheostomy	Blood<br>Catheter tip	Candidemia	FCZ<br>CFG	No	No	No	No	No	No
	Jyothi, L. et al., 2021 [57]	India	*C. kefyr*	M	51	NI	No	Diabetes mellitus<br>Hypertension<br>Diabetic retinopathy<br>CVA<br>Rhabdomyolysis<br>Acute kidney injury	Blood	Candidemia	FCZ	NI	Yes	Yes	NI	urinary tract infection	*Enterococcus faecalis*
	de Jong, A. W. et al., 2023 [36]	Malysia	*C. khanbhai*	M	55	14	Yes*	New-onset hospital-acquired pneumonia	Blood	Candidemia	NA	NI	No	No	No	No	No
	Fujita, S. et al., 2007 [54]	Japan	*C. nivariensis*	F	70	≥100	No	Rheumatic arthritis<br>Nutritional disorder	Blood	Candidemia	VCZ<br>MFG	NI	No	No	Yes	No	No
**Europe**	Ruiz Gaitán, A. C. et al., 2017 [37]	Spain	*C. auris*	M	26	56	No	Decompressive craniectomy and maxillo-facial emergencysurgery	Blood<br>Catheter tip	Candidemia	AFG	No	NI	No	No	No	No
			C. auris	M	48	44	No	Emergent decompressive craniectomy	Blood<br>Catheter tip	Candidemia	MFG	Yes	NI	No	No	No	No
			C. auris	M	66	84	Yes	Hepatocellular carcinoma<br>Liver resection	Blood<br>peritoneal fluid	Candidemia	AMB<br>AFG	Yes	Yes	NI	NI	No	No

Author/Year	Country	Candida species	G	Age (years)	Hospital stay (days)	Death	Comorbilities	Site of isolation	Subsequent infection type	Antifungal treatment	MV	IC	Previous broad-spectrum ATB	PAT	Other infections	Microorganism
		C. auris	F	39	91	Yes	Severe ventricular dysfunction	Blood<br>Catheter tip	Candidemia	FCZ	NI	No	Yes	Yes	No	No
Vogelzang, E. H. et al., 2019[45]	Netherlands	*C. auris*	M	middle-aged	NI	No	SEPSIS recently due to pneumonia	Catheter tip	No symptoms	No	Yes	Yes	Yes	Yes	No	No
Dewaele, K. et al., 2020 [44]	Belgium	*C. auris*	F	middle-aged	≥100	No	Bariatric surgery	Blood	Candidemia	AFG	Yes	No	Yes	No	ITU	*Klebsiella pneumoniae*<br>*Escherichia coli*
Katsiari, M. et al., 2023 [41]	Greece	*C. auris*	M	78	20	Yes	Diabetes mellitus<br>Arterial hypertension<br>Stent in the common bile duct	Blood	Candidemia	CFG<br>AMB	NI	Yes	No	No	No	No
Levy, Y. etr al., 2021 [43]	France	*C. auris*	M	36	19	Yes	Paroxysmal nocturnal hemoglobinuria<br>Cerebral thrombophlebitis	Blood<br>Catheter tip	Candidemia	CFG<br>AMB	NI	Yes	No	No	Endocarditis	*C. auris*
Desnos-Ollivier, M. et al., 2021 [48]	Greece	*C. auris*	M	54	50	Yes	Hepatitis C<br>Liver transplantation<br>Cholestasis	Hepatic drainage	Hepatic abcess	CFG<br>PCZ	NI	Yes	Yes	No	No	No
Seth-Smith, H. M. B. et al., 2020 [46]	Switzerland	*C. kefyr*	M	61	NI	No	Leukaemia<br>Allogeneic haematopoietic stem cell transplantation	Blood	Candidemia	FCZ<br>PCZ	NI	Yes	Yes	Yes	No	N/A
Spiliopoulou, A. et al., 2022 [40]	Greece	*C. kefyr*	M	41	50	No	Bronchial asthma<br>Severe Covid 19	Urine (+)<br>Blood (-)	Pyelonephritis	AMB<br>FCZ	Yes	No	NI	NI	No	No
Cartier, N. et al., 2020 [42]	France	*C. nivariensis*	M	77	51	No	Urothelial carcinoma<br>Cystoprostectomy<br>Primary pulmonary adenocarcinoma	Blood	Candidemia	CFG<br>VCZ	NI	Yes	Yes	Yes	No	No
Lopez-Soria, L. M. et al., 2013 [39]	Spain	*C. nivariensis*	M	81	56	No	Jejunocolic fistula<br>Severe malnutrition<br>Secondary anemia bilateral<br>Pulmonary thromboembolism	Blood	Candidemia	FCZ<br>CFG	NI	No	Yes	No	No	No

	Author/Year	*Country*	*Candida species*	*G*	*Age (years)*	*Hospital stay (days)*	*Death*	*Comorbidities*	*Site of isolation*	*Subsequent infection type*	*Antifungal treatment*	*MV*	*IC*	*Previous broad-spectrum ATB*	*PAT*	*Other infections*	*Microorganism*
	Musso, M. et al., 2014 [47]	Italy	*C. norvegensis*	M	47	30	No	HCV<br>Hepatocarcinoma<br>Transplant	Blood<br>Bile specimens	Candidemia<br>Intrabdominal abscesses	AFG	Yes	Yes	Yes	Yes	Candidemia<br>Bilateral pneumonia	*Enterococcus faecalis*<br>*Klebsiella pneumoniae*
	Sanclemente, G. et al., 2015 [38]	Spain	*C. norvegensis*	M	61	40	Yes	Hepatocellular carcinoma<br>Liver transplantation	Blood	Candidemia	AFG	NI	Yes	Yes	Yes	no	no
**Middle East**	Mohsin, J. et al., 2017 [33]	Oman	*C. auris*	M	77	40	No	Diabetes mellitus<br>Hypertension<br>Osteomyelitis	Blood	Candidemia	CFG	Yes	Yes	Yes	No	Candidemia	*Escherichia coli*
			C. auris	F	70	35	Yes	Diabetes mellitus<br>Arterial hypertension	Blood	Candidemia	AFG	NI	Yes	Yes	No	Candidemia<br>Gastric infection	*Enterococcus faecium*<br>*Helicobacter pylori*
	Al-Obaid, I. et al., 2022 [58]	Kuwait	*C. auris*	F	32	79	Yes	Bilateral lung transplant<br>Diabetes mellitus type 1<br>Chronic transplant rejection<br>Respiratory failure	Blood<br>Tracheal aspirate	Candidemia	AMB<br>CFG<br>VCZ	Yes	Yes	Yes	No	Colitis	*Cytomegalovirus*
	Alatoom, A. et al., 2018 [64]	United Arab Emirates	*C. auris*	F	84	92	Yes	Chronic renal failure<br>Hemodialysis<br>Severe psoriasis<br>Chronic atrial fibrillation<br>Hypertension	Blood	Candidemia	CFG<br>AMB	NI	Yes	Yes	NI	No	No
	Kiraz, N. et al., 2009 [68]	Turkey	*C. norvegensis*	F	53	23	Yes	Leukaemia	Blood	Candidemia	AMB	NI	Yes	Yes	Yes	No	No

Legend: Gender (G); Female (F); Male (M); Mechanical ventilation (MV); Imunocompromised (IC); Previous Antifungal therapy (PAT); Amphotericin B (AMB); Fluconazole (FCZ); Voriconazole (VCZ); Itraconazole (ICZ); Posaconazole (PCZ); 5-Flucytosine (5-FC); Caspofungin (CFG); Anidulafungin (AFG); Micafungin (MFG); No information (NI); * Died before TSA.

Table S3: Risk of Bias Assessment

Study	Was the study question or objective clearly stated?	Was the study population clearly and fully described, including a case definition?	Were the cases consecutive?	Were the subjects comparable?	Was the intervention clearly described?	Were the outcome measures clearly defined, valid, reliable, and implemented consistently across all study participants?	Was the length of follow-up adequate?	Were the statistical methods well-described?	Were the results well-described?	Quality Rating
**A Report of *Candida blankii* Fungemia and Possible Endocarditis in an Immunocompetent Individual and the Review of Literature**	Yes	Yes	NA	Yes	Yes	Yes	Yes	NA	Yes	Good (7)
**An Unusual Case of *Candida kefyr* Fungemia in an Immunocompromised Patient**	Yes	Yes	NA	Yes	Yes	Yes	Yes	NA	Yes	Good (7)
**Bloodstream infection with *Candida kefyr/Kluyveromyces marxianus*: case report and draft genome**	Yes	Yes	NA	Yes	Yes	Yes	Yes	NA	Yes	Good (7)
**Breakthrough fungemia due to *Candida fermentati* with fks1p mutation under micafungin treatment in a cord blood transplant recipient**	Yes	Yes	NA	Yes	Yes	Yes	Yes	NA	Yes	Good (7)
***Candida auris* in critically ill patients: Emerging threat in intensive care unit of hospitals**	Yes	Yes	No	Yes	Yes	Yes	Yes	NA	Yes	Good (7)
***Candida bracarensis* bloodstream infection in an**	Yes	Yes	NA	Yes	Yes	Yes	Yes	NA	Yes	Good (7)

**immunocompromised patient**										
***Candida duobushaemulonii* sepsis and *Candida auris* co-isolation following hospitalisation in Vietnam**	Yes	Yes	NA	Yes	Yes	Yes	No	NA	Yes	Fair (6)
***Candida kefyr*, an uncommon but emerging fungal pathogen: Report of two cases**	Yes	Yes	NA	Yes	Yes	Yes	Yes	NA	Yes	Good (7)
***Candida khanbhai* sp. nov., a new clinically relevant yeast within the *Candida haemulonii* species complex**	Yes	Yes	No	Yes	Yes	Yes	Yes	NA	Yes	Good (7)
***Candida nivariensis*: Identification strategy in mycological laboratories**	Yes	Yes	NA	Yes	Yes	Yes	Yes	NA	Yes	Good (7)
***Candida norvegensis* fungaemia in a neutropenic patient with acute non-lymphoblastic leukaemia**	Yes	Yes	NA	Yes	Yes	Yes	Yes	NA	Yes	Good (7)
***Candida norvegensis* fungemia in a liver transplant recipient**	Yes	Yes	NA	Yes	Yes	Yes	Yes	NA	Yes	Good (7)
**Case Report: Emergence of *Candida auris* in the Indian Ocean Region**	Yes	Yes	Yes	Yes	Yes	Yes	Yes	NA	Yes	Good (8)
**Catheter-related candidemia caused by *Candida haemulonii* in a patient in long-term hospital care**	Yes	Yes	NA	Yes	Yes	Yes	Yes	NA	Yes	Good (7)
**Catheter-related fungemia due to fluconazole-resistant *Candida nivariensis***	No	Yes	NA	Yes	Yes	Yes	Yes	NA	Yes	Fair (6)
**Development of High-Level Echinocandin Resistance in a Patient With Recurrent *Candida***	Yes	Yes	NA	Yes	Yes	Yes	Yes	NA	Yes	Good (7)

***auris* Candidemia Secondary to Chronic Candiduria [1]**										
**Earliest case of *Candida auris* infection imported in 2007 in Europe from India prior to the 2009 description in Japan**	Yes	Yes	NA	Yes	Yes	Yes	Yes	NA	Yes	Good (7)
**Emergence of Clonally-Related South Asian Clade I Clinical Isolates of *Candida auris* in a Greek COVID-19 Intensive Care Unit**	Yes	Yes	No	Yes	Yes	Yes	Yes	NA	Yes	Good (7)
**Fatal Breakthrough Candidemia in an Immunocompromised Patient in Kuwait Due to *Candida auris* Exhibiting Reduced Susceptibility to Echinocandins and Carrying a Novel Mutation in Hotspot-1 of FKS1**	Yes	Yes	NA	Yes	Yes	Yes	Yes	NA	Yes	Good (7)
**First case of *Candida auris* infection in Belgium in a surgical patient from Kuwait**	Yes	Yes	NA	Yes	Yes	Yes	Yes	NA	Yes	Good (7)
**First case report of catheter-related fungemia by *Candida nivariensis* in the Iberian Peninsula**	Yes	Yes	NA	Yes	Yes	Yes	Yes	NA	Yes	Good (7)
**First report of a clinical isolate of *Candida haemulonii* in Brazil**	Yes	Yes	NA	Yes	Yes	Yes	Yes	NA	Yes	Good (7)
**First report of sporadic cases of *Candida auris* in Colombia**	Yes	Yes	NA	Yes	Yes	Yes	Yes	NA	Yes	Good (7)
**First three reported cases of nosocomial fungemia caused by *Candida auris***	Yes	Yes	Yes	Yes	Yes	Yes	Yes	NA	Yes	Good (8)
**Infections due to *Candida haemulonii*: species identification, antifungal**	Yes	Yes	No	Yes	Yes	Yes	Yes	NA	Yes	Good (7)

**susceptibility and outcomes**										
**Invasive *Candida kefyr* infection presenting as pyelonephritis in an ICU hospitalized COVID-19 patient: Case report and review of the literature**	Yes	Yes	NA	Yes	Yes	Yes	Yes	NA	Yes	Good (7)
**Invasive Candidiasis due to *Candida norvegensis* in a Liver Transplant Patient: Case Report and Literature Review**	Yes	Yes	NA	Yes	Yes	Yes	Yes	NA	Yes	Good (7)
**Liver abscess caused by *Candida haemulonii* var. vulnera. First case report in Peru**	Yes	Yes	NA	Yes	Yes	Yes	Yes	NA	Yes	Good (7)
**Nosocomial fungemia by *Candida auris*: First four reported cases in continental Europe**	Yes	Yes	Yes	Yes	Yes	Yes	Yes	NA	Yes	Good (8)
**Persistent candidemia despite appropriate fungal therapy: First case of *Candida auris* from the United Arab Emirates**	Yes	Yes	NA	Yes	Yes	Yes	Yes	NA	Yes	Good (7)
**The first cases of *Candida auris* candidaemia in Oman**	Yes	Yes	Yes	Yes	Yes	Yes	Yes	NA	Yes	Good (8)
**The first invasive *Candida auris* infection in Taiwan**	Yes	Yes	NA	Yes	Yes	Yes	Yes	NA	Yes	Good (7)
**The first Russian case of candidaemia due to *Candida auris***	Yes	No	NA	Yes	No	Yes	Yes	NA	Yes	Fair (5)
**The first two cases of *Candida auris* in the Netherlands**	Yes	Yes	Yes	Yes	Yes	Yes	Yes	NA	Yes	Good (8)
**Three cases of *Candida fermentati* fungemia following hematopoietic stem cell transplantation**	Yes	Yes	No	Yes	Yes	Yes	Yes	NA	Yes	Good (7)

**Transient fungemia caused by an amphotericin B-resistant isolate of *Candida haemulonii***	Yes	Yes	NA	Yes	Yes	Yes	Yes	Yes	Yes	Good (8)

Table S4: Identification results of uncommon *Candida* species isolates from patients with invasive infection.

Author/Year	Microorganism	VITEK®		API® AUX		Microscan®	MALDI-TOF	DNA sequencing
		Model	Result	Model	Result			
Biagi, M. J. et al., 2019 [65]	*C. auris*						*C. haemulonii*	*C. auris*
Parra-Giraldo, C. M. et al., 2018 [112]	*C. auris*					*C. albicans*	*C. auris*	*C. auris*
Das, S. et al., 2018 [61]	*C. auris*					*C. famata*		*C. auris*
	C. auris					*C. famata*		*C. auris*
	C. auris					*C. famata*		*C. auris*
Lee, W. G. et al., 2011 [35]	*C. auris*	VITEK® 2 system	*C. haemulonii*	API® 20C AUX	*R. glutinis*			*C. auris*
Tsai, Y. T. et al., 2022 [32]	*C. auris*						*C. auris*	*C. auris*
Vasilyeva, N. V. et al., 2018. [55]	*C. auris*						*C. auris*	*C. auris*
Levy, Y. et al., 2021 [43]	*C. auris*						*C. auris*	

Author/Year	Microorganism	VITEK®		API® AUX		Microscan®	MALDI-TOF	DNA sequencing
		Model	Result	Model	Result			
Desnos-Ollivier, M. et al., 2021 [48]	*C. auris*							*C. haemulonii* *C. auris*
Katsiari, M. et al., 2023 [41]	*C. auris*						*C. auris*	*C. auris*
Dewaele, K et al., 2020 [44]	*C. auris*	VITEK® 2 system	*C. haemulonii*				*C. auris*	*C. auris*
Ruiz Gaitán, A. C. et al., 2017 [37]	*C. auris*	VITEK® MS	*C. lusitaniae (78%)*	API® 20C AUX	*C. sake (99.8%)*			*C. auris*
	C. auris	VITEK® MS	*C. haemulonii*	API® 20C AUX	*C. sake (99.8%)*			*C. auris*
	C. auris	VITEK® MS	*No identification*	API® 20C AUX	*C. sake (99.8%)*			*C. auris*
	C. auris	VITEK® MS	*No identification*	API® 20C AUX	*C. sake (99.8%)*			*C. auris*
Vogelzang, E. H. et al., 2019 [45]	*C. auris*						*C. auris*	*C. auris*
Al-Obaid, I. et al., 2022 [58]	*C. auris*	VITEK® 2 system	*C. haemulonii*					*C. auris*

Author/Year	Microorganism	VITEK® Model	VITEK® Result	API® AUX Model	API® AUX Result	Microscan ®	MALDI-TOF	DNA sequencing
Alatoom, A. et al., 2018 [64]	*C. auris*	VITEK® 2 system	*C. haemulonii*				*C. auris*	
Mohsin, J. et al., 2017 [33]	*C. auris*			API® 20C AUX	*C. haemulonii*		*C. auris*	*C. auris*
	C. auris			API® 20C AUX	*C. haemulonii*		*C. auris*	*C. auris*
Kollu, V. S. et al., 2021 [52]	*C. blankii*	No information	No information	No information	No information	No information	No information	No information
Warren, T. A. Et al., 2010 [53]	*C. bracarensis*			API® 20C AUX	C. glabrata			
Xie, O. et al., 2020 [56]	*C. duobushaemulonii*	Vitek® MS	*C. duobushaemulonii (99%)*				no result in any matched patterns	*C. duobushaemulonii*
	C. duobushaemulonii	Vitek® 2 system	*C. duobushaemulonii*					
Konuma, T. et al., 2017 [59]	*C. fermentati*	VITEK® 2 system	*C. famata*					*C. fermentati*
Morita, K. et al., 2018 [34]	*C. fermentati*							*C. fermentati*
	C. fermentati							*C. fermentati*
	C. fermentati *C. famata*							*C. fermentati*

Author/Year	Microorganism	VITEK®		API® AUX		Microscan ®	MALDI-TOF	DNA sequencing
		Model	Result	Model	Result			
Kim, S. et al., 2011 [63]	*C. haemulonii*	VITEK® 2 system	*C. haemulonii*					*C. haemulonii*
Ruan, S. Y. et al., 2010 [60]	*C. haemulonii*	VITEK® 2 system	*C. haemulonii*	API® 32C system	*C. intermedia* or *C. sake* or *C. globosa* or *C. melibiosica* or *Saccharomyces kluyveri*			*C. haemulonii*
	C. haemulonii	VITEK® 2 system	*C. haemulonii*	API® 32C system	*C. sake*			*C. haemulonii*
Pérez-Lazo G. et al., 2021 [51]	*C. haemulonii*	VITEK® 2 system	*C. haemulonii*					
Almeida-Jr, J. N. et al., 2012 [49]	*C. haemulonii*	VITEK® 2 system	*C. haemulonii*					*C. haemulonii*
Rodero, L. et al., 2002 [50]	*C. haemulonii*	VITEK® system	*Pichia ohmeri*	API® 32C system	No identification		*C. haemulonii*	
Seth-Smith, H. M. B. et al., 2019 [46]	*C. kefyr*						*No identification*	*C. kefyr*
Jyothi, L. et al., 2021 [57]	*C. kefyr*					*C. kefyr (99.99)*		

Author/Year	Microorganism	VITEK®		API® AUX		Microscan ®	MALDI-TOF	DNA sequencing
		Model	Result	Model	Result			
Corpus, K. et al., 2004 [67]	*C- kefyr*				No information			
Spiliopoulou, A. et al., 2021 [40]	*C. kefyr*			API® 20C AUX	*C. kefyr*		*C. kefyr*	
de Jong, A. W. et al., 2022 [36]	*C. khanbhai*						*C. khanbhai*	
Fujita, S. et al., 2007 [54]	*C. nivariensis*							*C. nivariensis*
Cartier, N. et al., 2020 [42]	*C. nivariensis*			API® 20C AUX	*C. glabrata*		*C. nivariensis*	*C. nivariensis*
Lopez-Soria, L. M. et al., 2013 [39]	*C. nivariensis*	VITEK® 2 system	No conclusive identification					*C. nivariensis*
Sanclemente, G. et al., 2015 [38]	*C. norvegensis*						*C. norvegensis*	

Author/Year	Microorganism	VITEK®		API® AUX		Microscan®	MALDI-TOF	DNA sequencing
		Model	Result	Model	Result			
Musso, M. et al., 2014 [47]	*C. norvegensis*	VITEK® 2 system	*C. norvegensis*					
Kiraz, N. et al., 2009 [68]	*C. norvegensis*			API® 20C AUX	*C. norvegensis*			

Table S5: Minimal inhibitory concentrations (MIC; mg/L) and mutations of uncommon *Candida* spp. Isolated from patients with invasive infections.

Author/Year	Microorganisms	TSA Method	AMB	FCZ	VCZ	ICZ	PCZ	5-FC	CFG	AFG	MFG	Mutation
Biagi, M. J. et al., 2019 [65]	*C. auris*	BM CLSI Sensititre YeastOne®	1	2	0.015	≤0.03	ND	ND	0.06	0.12	0.12	*FKS*1 (S639P)
Parra-Giraldo, C. M. et al., 2018 [112]	*C. auris*	Etest®	0.75	24	0.64	0.25	0.023	ND	0.47	0.12	0.19	NP
Das, S. et al., 2018 [61]	*C. auris*	Etest®	1	8	0.125	0.064	0.047	ND	0.25	ND	ND	
	C. auris	Etest®	8	64	0.094	0.064	0.125	ND	0.023	ND	ND	
	C. auris	Etest®	16	8	0.19	0.047	0,064	ND	0,25	ND	ND	
Al-Obaid, I. et al., 2022 [58]	*C. auris*	MICRONAUT® system	1	≥128	0.5	0.5	0.063	ND	ND	0.25	0.125	*FKS*1 (S639Y) (S639T) *ERG*11 (K143R)

Author/Year	Microorganisms	TSA Method	AMB	FCZ	VCZ	ICZ	PCZ	5-FC	CFG	AFG	MFG	Mutation
Lee, W. G. et al., 2011 [35]	*C. auris*	BM CLSI	1	8	0.06	0.25	ND	ND	0.06	ND	0.03	NP
Tsai, Y. T. et al., 2022 [32]	*C. auris*	BM CLSI	1	8	0.12	ND	0.12	≤ 0.06	ND	0.12	0.06	*ERG*11 and *FKS*1 not detected
Vasilyeva, N. V. et al., 2018. [55]	*C. auris*	BM CLSI	0.5	≥ 128	≥ 8	ND	≥ 2	ND	≥ 4	ND	ND	NP
Levy, Y. et al., 2021 [43]	*C. auris*	BM EUCAST	0.25	≥64	1	ND	≤0.014	≥64	0.015	ND	0.03	
Desnos-Ollivier, M. et al., 2021 [48]	*C. auris*	BM EUCAST	0.5	≥64	0.5	ND	0.125	NA	0.06	ND	0.5	
Katsiari, M. et al., 2023 [41]	*C. auris*	BM EUCAST	2	>128	0.12	0.06	0.12	0.12	ND	0.25	0.25	
Dewaele, K et al., 2020 [44]	*C. auris*	BM EUCAST	0.5	>64	> 4	> 4	> 4	0.25	ND	0.125	≤0.03	

Author/Year	Microorganisms	TSA Method	AMB	FCZ	VCZ	ICZ	PCZ	5-FC	CFG	AFG	MFG	Mutation
2020	*C. auris*	Sensititre YeastOne®	1	256	8	16	8	0,125	NA	0,125	0,125	
Ruiz Gaitán, A. C. et al., 2017 [37]	*C. auris*	Sensititre YeastOne®	0.5	≥256	2	ND	ND	≤0.06	ND	ND	ND	
	C. auris	Sensititre YeastOne®	0.5	≥256	2	ND	ND	≤0.06	ND	ND	ND	
	C. auris	Sensititre YeastOne®	0.5	≥256	2	ND	ND	≤0.06	ND	ND	ND	
	C. auris	Sensititre YeastOne®	0.5	≥256 mg/L	2	ND	ND	≤0.06	ND	ND	ND	
Vogelzang, E. H. et al., 2019 [45]	*C. auris*	BM CLSI	0.5	> 64	4	ND	ND	ND	ND	<0.063	0.063	NP
Alatoom, A. et al., 2018 [64]	*C. auris*	No information	1	ND	1	ND	ND	ND	0.25	NA	NA	
Mohsin, J. et al., 2017 [33]	*C. auris*	BM CLSI	2	64	0.125	0.031	<0.016	ND	ND	0.031	0.063	
	C. auris	BM CLSI	1	>64	1	0.0125	0.063	ND	ND	0.125	0.125	

Author/Year	Microorganisms	TSA Method	AMB	FCZ	VCZ	ICZ	PCZ	5-FC	CFG	AFG	MFG	Mutation
Kollu, V. S. et al., 2021 [52]	*C. blankii*	No information	0.500	16	0.250	0.500	1	≤0.060	1	0.250	0.120	
Warren, T. A. Et al., 2010 [53]	*C. bracarensis*	Sensititre YeastOne®	0.25	8	0.12	0.25	0.5	≤ 0.06	0.06	0.03	≤ 0.008	
Xie, O. et al., 2020 [56]	*C. duobushaemulonii*	Sensititre YeastOne®	0.5	64	ND	ND	ND	ND	ND	ND	0.06	
Konuma, T. et al., 2017 [59]	*C. fermentati*	No information	1	4	0.12	1	NA	≤ 0.12	NA	NA	1	*FKS*1 (L633M) (T634A)
Morita, K. et al., 2018 [34]	*C. fermentati*	BM CLSI	0.5	8	ND	1	ND	≤ 0.12	ND	ND	0.5	
	C. fermentati	BM CLSI	0.25	4	ND	1	ND	≤ 0.12	ND	ND	1	
	C. fermentati *C. famata*	BM CLSI	0.25	8	ND	0.5	ND	≤ 0.12	ND	ND	0.5	
Almeida-Jr, J. N.	*C. haemulonii*	Sensititre YeastOne®	4	8	0.064	0.25	0.125	64	0.25	ND	ND	

Author/Year	Microorganisms	TSA Method	AMB	FCZ	VCZ	ICZ	PCZ	5-FC	CFG	AFG	MFG	Mutation
et al., 2012 [49]												
Pérez-Lazo G. et al., 2021 [51]	*C. haemulonii*	BM CLSI	1	> 64	ND	ND	ND	ND	ND	0.06	ND	
Rodero, L. et al., 2002 [50]	*C. haemulonii*	BM CLSI with EUCAST modification	4	32	0.12	ND	ND	0.12	ND	ND	ND	NP
Kim, S. et al., 2011 [64]	*C. haemulonii*	No information	0.5	8	0.5	0.25	ND	ND	0.125	ND	ND	
Ruan, S. Y. et al., 2010 [60]	*C. haemulonii*	BM CLSI	2	16	0.25	0.25	0.12	0.06	1	0.25	0.12	
	C. haemulonii	BM CLSI	2	16	0.25	0.25	0.12	0.12	1	0.12	0.12	
Corpus, K. et al., 2004 [67]	*C. kefyr*	Macrodilution CLSI 24h	0.125	0,25	0.03	0.06	ND	ND	≤ 0.125	ND	ND	
	C. kefyr	Macrodilution CLSI 48h	0,25	0,5	0,03	0,06	ND	ND	≤ 0.125	ND	ND	
Jyothi, L. et al., 2021 [57]	*C. kefyr*	Etest®	S	S	S	S	ND	ND	ND	ND	ND	

Author/Year	Microorganisms	TSA Method	AMB	FCZ	VCZ	ICZ	PCZ	5-FC	CFG	AFG	MFG	Mutation
Seth-Smith, H. M. B. et al., 2019 [46]	*C. kefyr*	Sensititre YeastOne®	1	0.12	≤0.008	0.12	0.06	0.5	≤ 0.08	0.03	0.03	
Spiliopoulou, A. et al., 2021 [40]	*C. kefyr*	BM EUCAST	2	0.12	0.008	0.06	0.06	1	0.03	0.12	0.12	
	C. kefyr	Etest®	>32	0.38	0.023	0.125	0.126	2	0.25	0.064	0.047	
de Jong, A. W. et al., 2022 [36]	*C. khanbhai*	BM EUCAST 24h	2	>64	0,5	1	2	4	ND	0,5	0,25	
	C. khanbhai	BM EUCAST 48h	4	>64	16	16	4	16	ND	1	0,5	
Fujita, S. et al., 2007 [54]	*C. nivariensis*	ASTY® microdilution	0.5	ND	4	≥128	ND	2	ND	ND	0.06	
Cartier, N. et al., 2020 [42]	*C. nivariensis*	BM EUCAST	0,06	4	0,25	NA	0,125	ND	ND	ND	≤0.08	
	C. nivariensis	Etest®	0,75	6	0,047	NA	0,38	ND	ND	ND	0,012	
Lopez-Soria, L. M.	*C. nivariensis*	Sensititre YeastOne®	1	4	0.03	0.25	0.25	0.125	0.125	0.015	0.015	NP

Author/Year	Microorganisms	TSA Method	AMB	FCZ	VCZ	ICZ	PCZ	5-FC	CFG	AFG	MFG	Mutation
et al., 2013 [39]												
Sanclemente, G. et al., 2015 [38]	*C. norvegensis*	BM CLSI	0,5	64	0,75	4	ND	64	0,047	ND	ND	
Musso, M. et al., 2014 [46]	*C. norvegensis*	Vitek® 2 system	NA	8	0.25	ND	ND	ND	ND	ND	ND	
Kiraz, N. et al., 2009 [68]	*C. norvegensis*	BM CLSI	0.25	128	ND	0,5	ND	ND	ND	ND	ND	

Legend: Broth Microdilution (BM); Amphotericin B (AMB); Fluconazole (FCZ); Voriconazole (VCZ); Itraconazole (ICZ); Posaconazole (PCZ); 5-Flucytosine (5-FC); Caspofungin (CFG); Anidulafungin (AFG); Micafungin (MFG); European Committee on Antimicrobial Susceptibility Testing (EUCAST); Clinical & Laboratory Standards Institute (CLSI); Not determined (ND); Susceptible (S); Not performed (NP); Serine (S); Proline (P); Threonine (T); Leucine (L); Alanine (A); Tyrosine (Y); Methionine (M); Arginine (R); Lysine (K).
